# A study of impulsivity and adverse childhood experiences in a population health setting

**DOI:** 10.3389/fpubh.2024.1447008

**Published:** 2024-12-04

**Authors:** Robert W. Read, Karen A. Schlauch, Gai Elhanan, Iva Neveux, Stephanie Koning, Takesha Cooper, Joseph J. Grzymski

**Affiliations:** ^1^Department of Internal Medicine, School of Medicine, University of Nevada, Reno, Reno, NV, United States; ^2^Department of Health Behavior, Policy, and Administrative Sciences, School of Public Health, University of Nevada, Reno, Reno, NV, United States; ^3^Renown Health, Reno, NV, United States; ^4^Department of Psychiatry and Behavioral Sciences, School of Medicine, University of Nevada, Reno, Reno, NV, United States

**Keywords:** public mental health, impulsivity, adverse childhood experiences, social determinants of health, PheWAS, Kaplan–Meier analyses

## Abstract

As complex mental health traits and life histories are often poorly captured in hospital systems, the utility of using the Barratt Impulsivity Scale (BIS) and Adverse Childhood Experiences (ACEs) for assessing adult disease risks is unknown. Here, we use participants from the Healthy Nevada Project (HNP) to determine if two standard self-assessments could predict the incidence and onset of disease. We conducted a retrospective cohort study involving adult participants who completed the Behavioral and Mental Health Self-Assessment (HDSA) between September 2018 and March 2024. Impulsivity levels were measured using the BIS-15, and retrospective self-reports of ACEs were collected through a standardized questionnaire. In total, 17,482 HNP participants completed the HDSA. Our findings indicate that ACEs were significantly associated with impulsivity. Disease associations with impulsivity and ACEs were evaluated using a phenome-wide association study, identifying 230 significant associations with impulsivity. Among these, 44 were related to mental health diagnoses, including major depressive disorder (MDD). Kaplan–Meier survival estimates characterized the differences in the lifetime predicted probability between high and low impulsivity for major depressive disorder and essential hypertension. This analysis showed that having both high ACEs and high impulsivity confer substantial risk of MDD diagnosis (hazard ratios 2.81, 2.17, respectively). Additionally, lifetime predicted probability of MDD was approximately 40% higher for high ACEs and high impulsivity compared to no ACEs and low impulsivity. Essential hypertension demonstrated similar trends, with an approximate 20% increase in predicted lifetime probability of diagnosis. These results demonstrate that high ACES and elevated impulsivity scores are associated with a range of negative health outcomes and a simple self-assessment of complex traits and life history may significantly impact clinical risk assessments.

## Introduction

Defining the causes associated with health outcomes of any individual is complex and requires an examination and understanding of clinical diagnoses, lab values, genetics, environmental exposures and life history. A specific trait may take on a different meaning in a life history marked by trauma or mental health disorders. For example, individuals diagnosed with mental health disorders often face a heightened risk of mortality compared to those without such conditions (1–3). Poor individual mental health is associated with a diminished quality of life (4–7) and an increased likelihood of substandard physical health. This results in decreased individual productivity, decreased self-care, and increased healthcare costs.

Population genomic health studies such as the Healthy Nevada Project (HNP) (8), Geisinger’s MyCode (9), and NIH’s All of US (10), inform participants of specific inherited health risks (e.g., CDC Tier 1 conditions), but most of these programs do not return disease risks approximated by non-genetic factors. In light of Nevada’s historically poor health outcomes and healthcare infrastructure (11, 12), the HNP strives to generate a comprehensive picture of health status and associated risks among its participants. To better understand the impact of health determinants, the HNP collects broader behavioral, Adverse Childhood Events (ACEs), quality of life, and mental/physical health phenotype data through the self-reported HDSA.

Impulsivity is defined as a lack of inhibition towards engaging in increasingly risky behavior, with little consideration for long-term negative consequences (13, 14). Risky behaviors include, but are not limited to, driving violations (15), high risk sexual behaviors (16), substance use and drug experimentation (17), and gambling (18). Impulsivity is also positively associated with several psychiatric and somatic disorders, including attention deficit hyperactivity disorder (ADHD) (19, 20), bipolar disorder (21), schizophrenia (22), cardiovascular disease (23), eating disorders (24) and is negatively associated with obsessive compulsive disorder (OCD) (25). Many previous studies show that impulsive behavior contributes to poor physical and mental health, shortened lifespan, and may result in approximately 6 trillion dollars in lost productivity worldwide by 2030 (26).

Additionally, previous studies (27–29) established a connection between impulsive behavior and childhood trauma, particularly adverse childhood experiences (ACEs). ACEs, commonly assessed using a standard 10-item retrospective recall questionnaire (30), include various forms of childhood maltreatment: emotional, physical, sexual abuse, neglect, substance abuse within the household, mental illness in the household, household domestic violence, parental separation and incarceration of a household member (7, 30). Factors like ACEs, poor quality of life, and impulsive behavioral traits often occur more frequently in households with lower socioeconomic status (7, 31). The HNP has collected 20,000 participants’ childhood events since 2018. Studies indicate that approximately 63% of the general population has experienced at least one ACE before age 18 (32, 33), and these childhood experiences are associated with negative health outcomes later in life, including notable increased prevalence of severe mental disorders such as suicidal ideation, major adult depression, and schizophrenia (7, 30, 34, 35). In the HNP, 66% of participants reported at least one ACE; HNP participants with four or more ACEs were nine times more likely to try to commit suicide. In this study, we leverage the HDSA to explore associations between impulsivity and other diseases in the HNP, concurrent with measuring the risk of high impulsivity and high ACEs on lifetime disease probability for essential hypertension and MDD. We hypothesize that social health determinants, such as ACEs and the Barratt Impulsivity Scale may act as accessible, non-invasive, low-cost predictors of specific mental and physical health disorders. These assumptions are based on a number of previous studies that link ACEs, quality of life scores, and impulsivity to health outcomes (25, 30, 33, 34, 36–39).

## Materials and methods

### Participants: the healthy Nevada project

The Healthy Nevada Project is an all-comers volunteer community population health study initiated in 2016 in Northern Nevada (7, 8, 38, 40–42). The current Healthy Nevada Project (HNP) includes 53,500 participants with 82% cross-referenced to EHR data. This study focuses on a subset of the HNP: 17,482 participants who provided responses to both the Barratt Impulsivity Scale and ACE questions contained in the Behavioral and Mental Health Self-Assessment (HDSA). No other selection criteria were used. This subcohort is denoted HNP_BIS_; its demographics are provided in Table 1. Sex was self-reported and classified as Male/Female. Ethnicity was based on genetic similarity and reported as: African, East Asian, European, Latinx, Other, South Asian.

**Table 1:** Demographics of 17,482 HNP_BIS_ participants.

Demographic characteristic	HNP_BIS_
Age at consent, mean, (sd), years	51.54 (16.18)
No. (%) Female	12,458 (71.26)
No. (%) Male	5,024 (28.74)
BIS-15, mean, (sd)	27.74 (6.42)
ACE score, mean, (sd)	2.13 (2.33)
No. (%) African	324 (0.02)
No. (%) East Asian	413 (0.03)
No. (%) European	14,134 (80.85)
No. (%) Latinx	1,962 (0.11)
No. (%) Other	581 (0.03)
No. (%) South Asian	68 (0.004)

### Measurements: the behavioral and mental health self-assessment questionnaire

Each individual who consents to participate in the HNP is also invited to complete the HDSA, which is administered by the Survey Monkey® platform. This questionnaire contains 103 questions that cover a wide range of behavioral and mental characteristics, such as ACEs, quality of life, impulsivity tendencies, incidence of mental illness and substance use. The 10-item ACE questionnaire follows the standard format used in previous studies (7, 29, 30, 36, 38, 39). Individual impulsivity is measured via the short-form Barratt-15 impulsivity scale (BIS-15) (43). Both ACE and BIS-15 questions are contained in the HDSA. Each ACE event is scored based on a “Yes” or “No” response with unanswered questions left as “NA.” The final composite ACE score is a sum of “Yes” responses for each participant (7, 38, 44). Only participants who answered all ten ACE questions were included in this study. The ACE score (a measure between 0 and 10), does not measure frequency of any ACE event; instead, it measures the number of different ACEs a participant has encountered. We define the “high ACE” subcohort as participants with four or more ACEs, and “low ACE” subcohort as those with zero ACEs (7, 33, 38, 45).

The Barratt Impulsiveness Scale (BIS-11) is a standard 30-item questionnaire that measures an individual’s impulsivity. Each question is answered on a four-point scale: 1 = rarely/never; 2 = occasionally; 3 = often; 4 = almost always/always. The BIS-11 Score is calculated as the sum of the 30 answers: greater scores indicate higher levels of impulsive behavior. Meule adapted this to a shorter 15-item version, the B1S-15, which performs comparably to longer questionnaires common in all-comers population health studies (43, 46, 47). The standard form is included in Supplementary Table S1. Only participants who answered all 15 questions of the BIS-15 were included in this study: no values were imputed. The range of the BIS-15 impulsivity score is [15, 60], but there is no consensus of what thresholds discriminate between “impulsive” behavior and “non-impulsive” behavior. For this study, we used the 80th percentile (33) to separate the participants into “high Impulsivity” and “low Impulsivity” cohorts.

### Phenome-wide association data analysis

Phenome-wide association studies (pheWAS) examine and identify associations between a large number of disease phenotypes and a specific genotype or phenotype of interest. The pheWAS approach examines associations between 1,252 possible disease phenotypes in the HNP cohort and BIS-15 levels, adjusting for ACEs. Disease phenotypes are based on participants’ recorded EHR ICD codes, which are then aggregated and converted into 1,252 individual phenotype groups (“phecodes”) using the R package **pheWAS** as described in Carroll and Denny (48, 49). Complete pheWAS methods are included in the Supplementary Methods.

### Disease-free survival data analysis

Kaplan–Meier survival analysis was conducted to estimate disease-free survival probabilities over time (50). The restricted mean survival time (RMST) was determined using the **survival** package in R. Sex, ACEs, and BIS-15 levels were considered as covariates. Differences in survival curves were compared using a log-rank test. Hazard ratios were calculated using the Cox Proportional Hazards model in R. The Schoenfeld residuals test applied to the MDD model indicates that the hazards of ACEs and impulsivity are proportional over time. However, the Schoenfeld residuals test applied to essential hypertension indicated there were non-proportional hazards for ACEs and impulsivity. To mitigate this, we stratified the hazards of ACEs and impulsivity into three empirically determined age groups (<=25, 26–60, >60). After stratification, the Schoenfeld residuals test was rerun and indicated proportional hazards within each of these groups over time.

## Results

### Demographics of impulsivity and ACEs in the HNP

In March 2024, a total of 17,482 HNP participants (age at consent 51.54 [16.19] years; 12,458 Female [71.26%]; African [1.85%], East Asian [2.36%], European [80.85%], Latinx [11.22%], Other [3.32%], South Asian [0.39%]) were assigned a BIS-15 score based on the 15 impulsivity-related questions, and an ACE score formulated on 10 ACE questions (Table 1). Incomplete answers were not imputed, and only participants with a 100% response rate were included with respect to both self-assessments. Based on previous work, ACEs and impulsivity are dependent events in the HNP_BIS_ (7). A Fisher exact test performed on the sub-cohorts of High/Low Impulsivity and High/Low ACEs yielded an odds ratio of 3.56 (*p* < 2.2 ×10^−16^) (Supplementary Table S2).

### Phenome-wide analysis (pheWAS) with ACEs and the Barratt impulsivity scale

**Figure 1:**
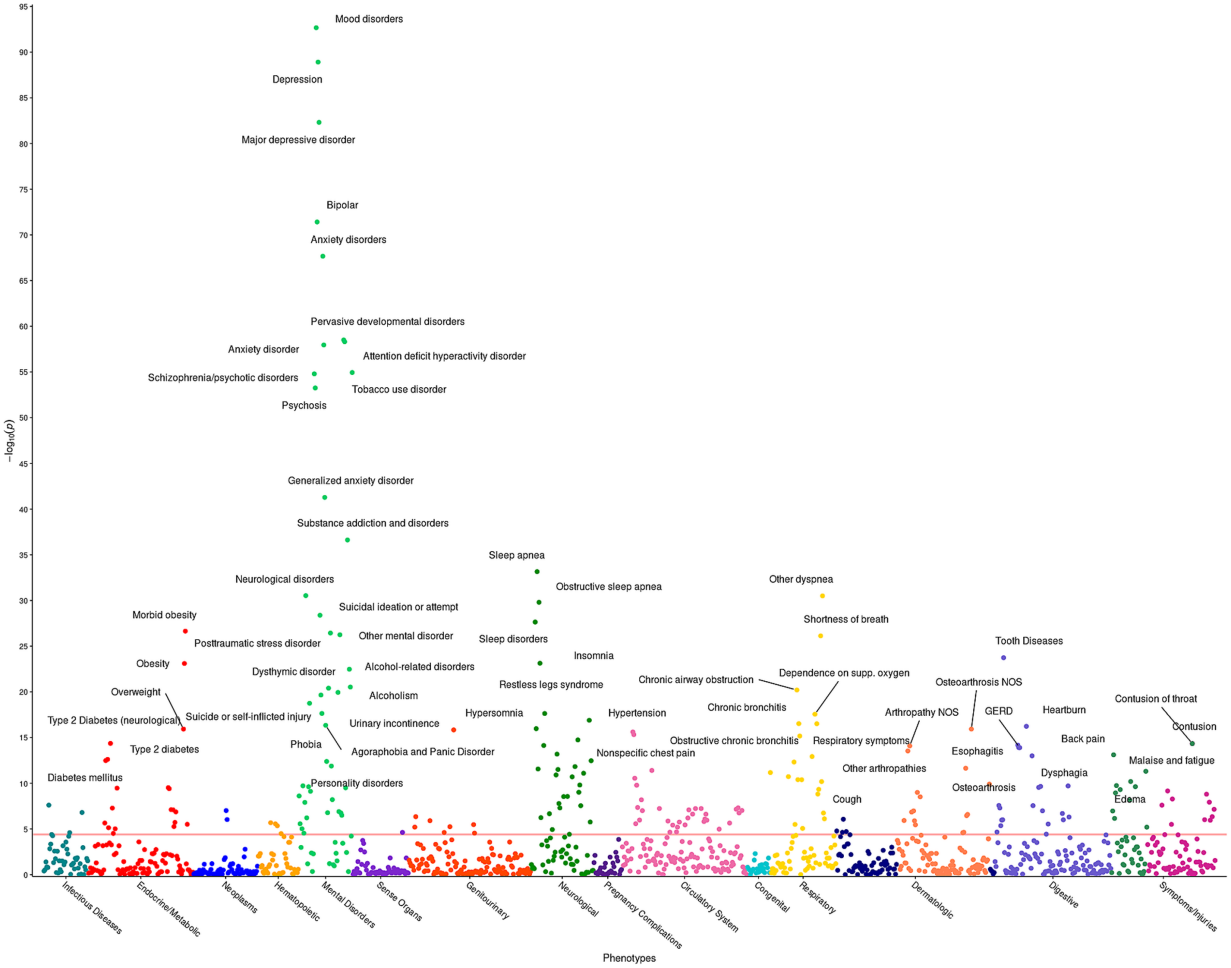
Impulsivity pheWAS based on BIS-15 score. This figure illustrates the results of 1,252 individual associations between the BIS-15 score and the incidence of phenotype group (phecode) of the HNP_BIS_ cohort. Covariates included in the models are sex, age at consent, and the ACE score. Each point denotes the *p*-value of association of that phenotype. The red horizontal line represents the Bonferroni-corrected significance level of *α* = 0.05/1,252 = 4 × 10^−5^. Only associations with *p* < 1 × 10^−10^ are annotated for ease of viewing.

The pheWAS determines whether any of the 1,252 impulsivity - disease associations are statistically significant. Given the significant association observed between ACEs and impulsivity, the model incorporated ACEs as a covariate with age and sex. Supplementary Table S3 presents the 230 statistically significant associations between diseases and the BIS-15 for fixed ACE score. Out of these 230 significant phenotypes, 44 are mental health related, including MDD, suicide ideation, ADHD, bipolar disorder, and schizophrenia (Figure 1). Additionally, strong associations with essential hypertension, obesity, sleep, pain, and respiratory disorders were identified.

### Risk of impulsivity and ACEs on the probability of major depressive disorder and essential hypertension diagnosis

**Figure 2:**
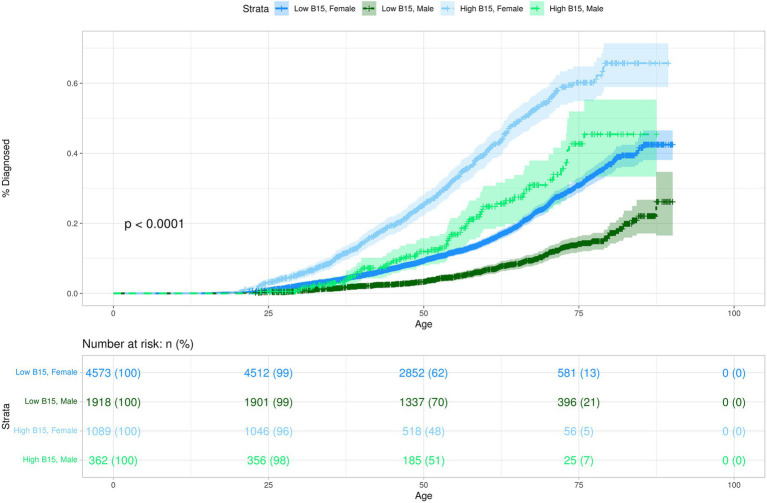
This Kaplan–Meier plot demonstrates the predicted probability of MDD diagnosis over time for participants stratified by impulsivity and sex. The *y*-axis represents the probability of being diagnosed with disorder. The *x*-axis represents participant age in years. Differences between the groups are visually depicted by the separation between the curves, indicating the relative rate at which events occur within each group. The shaded areas around each curve represent the 95% confidence intervals for the cumulative event estimates.

Given the associations observed in the phenome-wide analysis between impulsivity, MDD, and essential hypertension, we conducted Kaplan–Meier survival analysis to explore the potential impacts of high impulsivity and adverse childhood experiences (ACEs) on these two disorders. This resulted in an overall subcohort size of 7,944 participants. When stratified strictly by impulsivity, restricted mean survival times (RMST) for MDD were approximately 12 years shorter between those with high impulsivity and those with low impulsivity (Supplementary Table S4). When stratified solely by ACEs, the results for MDD were similar to impulsivity; an approximate 13-year difference in RMST (Supplementary Table S5). Furthermore, Cox proportional hazards models illustrate that both higher impulsivity and higher ACEs were associated with an increased risk of MDD (Impulsivity: HR 3.01; 95% CI, 2.69–3.37; *p* = 9.07 × 10^−83^ – ACEs: HR 3.56; 95% CI, 3.20–3.99; *p* = 1.29 × 10^−116^) (Supplementary Table S6). Given the higher likelihood of MDD development among females compared to males (51), we subsequently adjusted our model for sex. Overall prevalence rates for MDD in the HNP_BIS_ are greater in individuals with higher impulsivity scores, across both male and female cohorts (Figure 2 and Table 2). RMST for females were lower for both high and low impulsivity groups in comparison to the males (Supplementary Table S7), and the hazard ratio for sex shows that males have approximately 50% of the risk of being diagnosed for MDD (Figure 2 and Supplementary Table S6). Even after the adjustment for sex, hazard ratios for both high impulsivity and high ACEs were still markedly significant (Supplementary Table S6 and Supplementary Figure S1).

**Table 2:** Probability of disease stratified by sex for MDD and essential hypertension.

Outcome	Disorder	Sex	*N*	Probability at age 50 (%)	Probability at age 60 (%)	Probability at age 70 (%)	Lifetime probability (%)
HA and HB	MDD	F	783	29.35	46.86	62.9	72.49
		M	201	14.73	30.05	39.00	47.41
NA and LB	MDD	F	2,709	5.44	8.3	14.60	24.62
		M	1,454	1.92	4.00	7.58	9.76
HA and HB	ET	F	783	22.14	45.59	71.99	87.88
		M	201	29.12	52.07	82.14	82.16
NA and LB	ET	F	2,709	7.91	19.94	46.56	70.55
		M	1,454	9.56	24.08	54.45	65.23

LB, low BIS; HB, high BIS; M, male; F, female; NA, no ACEs; HA, high ACEs; MDD, major depressive disorder; ET, essential hypertension.

Incorporating ACEs, impulsivity, and sex into the same model allowed us to estimate the relative hazard ratios for each factor while adjusting for the influence of the others. Both high impulsivity and high ACEs were still strongly associated with MDD, even when accounting for the effects of the other (Impulsivity: HR 2.17; 95% CI, 1.93–2.43; *p* = 1.78×10^−39^ – ACEs: HR 2.81; 95% CI, 2.51–3.14; *p* = 5.08×10^−72^) (Supplementary Table S6). The combined RMST for MDD showed that females had an approximate 20-year difference in RMST between those with high ACEs and high impulsivity and those with no ACEs and low impulsivity (Supplementary Table S8). This was consistent in males with an approximate 14-year difference; however, the size of the male cohort with high ACEs and high impulsivity was small (Supplementary Table S8). The model also allowed us to predict the probability of MDD diagnosis comparing the best and worst outcomes. The CDC estimates that the life expectancy for Nevada is approximately 73 years for men and 79 years for women (52). These ages were used to calculate the predicted lifetime probability of being diagnosed with MDD under poor outcomes as well as under more positive outcomes in the HNP_BIS_. The predicted lifetime probability of being diagnosed with MDD for females with no ACEs and low impulsivity was 24.6% (Table 2). This is slightly under the women’s national average reported by Hasin et al. in 2018 (53). However, for the 783 females with high ACEs and high impulsivity, the predicted lifetime probability of being diagnosed with MDD was 72.5%. While males generally have lower predicted lifetime probabilities of diagnosis for both outcomes, a substantial difference in predicted probabilities persists between the two outcomes: no ACEs and low impulsivity – 9.76%, high ACEs and high impulsivity (*N* = 201)–47.41% (Table 2). In this cohort, there are a low number of events for males with high impulsivity and high ACEs, thus the results pertaining to the males should be taken with caution until a larger cohort is established.

Results from essential hypertension follow a similar pattern to MDD; however, we observed that the hazard ratios were considered non-proportional based on the Schoenfeld residuals test. Therefore, to account for any bias that may arise from this, we stratified by three age groups (<=25, 26–60, >60). Following stratification, the Schoenfeld residuals test was rerun and determined that the hazard ratios for ACEs and impulsivity were proportional within those three age groups. For participants less than 25, the hazard ratios for ACES and impulsivity were not significant; the small number of participants in that group with an essential hypertension diagnosis likely produced biased results. However, between 26 and 60, as well as those older than 60, the hazard ratios for impulsivity and ACEs were relatively consistent, although considerably lower than that hazard ratios seen for MDD (Impulsivity: HR[26–60]: 1.29, *p*[26–60] = 3.98×10^−05^; HR[>60]: 1.23, *p*[>60] = 1.80×10^−02^ – ACEs: HR[26–60]: 1.21, *p*[26–60] = 6.69×10^−04^; HR[>60]: 1.29, *p*[>60] = 2.56×10^−05^) (Supplementary Table S6). Moreover, in contrast to MDD, males are at an increased risk for essential hypertension (HR[26–60]: 1.30, *p*[26–60] = 2.86×10^−06^; HR[>60]: 1.26, *p*[>60] = 4.12×10^−05^), consistent with previous research (Supplementary Figures S2, S3) (54). RMST for males was lower than females for both high impulsivity and high ACEs compared to controls (Supplementary Tables S9, S10); however, there was no significant difference in overall prevalence between the impulsivity and ACE groups. The combined model for essential hypertension showed that both females and males had an approximate 9-year difference in RMST for participants with no ACEs and low impulsivity compared to those with high ACEs and high impulsivity (Supplementary Table S11). Furthermore, the lifetime probability of being diagnosed with essential hypertension for females with no ACEs and low impulsivity was approximately 70.6%. According to the NIH, approximately 75% of those over 70 years old are affected with hypertension, consistent with our findings (55). Those females with high ACEs and high impulsivity had a predicted lifetime probability of 87.9%. The males with low impulsivity and no ACEs demonstrated a predicted lifetime probability of 65.2% compared to 82.1% for those with high ACEs and high impulsivity. While males may have an overall higher risk for hypertension during their lifetime, the rate at which females develop hypertension increases after age 60, possibly accounting for the overall lifetime higher predicted probabilities we observed in the females (56).

## Discussion

Self-assessments such as the BIS-15 and ACE scores are not well-captured by clinical encounters and are shown here to be effective tools to investigate health outcomes. There are many explanations for this, including patients seeking behavioral and mental health care separately from somatic care to clinician inability of capturing behavioral traits and life histories during short visits. The HNP augments electronic health information with self-assessments to overcome some of these limitations. In doing so, we can leverage the tertiary care health systems’ examination of patients with a wide range of health problems through models incorporating both healthcare information and self-reported data. Simple self-assessments may serve as a valuable mental health diagnostic tool for large hospital systems focused on acute and primary care.

The relationship between ACEs and impulsive behavior is well-documented yet unsurprising: those with more childhood trauma present with less impulse control as adults (27–29, 57–59). This specific relationship was replicated in the HNP_BIS_, as shown in Supplementary Table S2. Overall, the pheWAS demonstrates that BIS-15 scores strongly associate with several clinically relevant mental health diagnoses including ADHD, which was one of the most anticipated associations (19). Impulsivity is also a key feature of several psychiatric disorders (14), and has been associated with many of them previously (19–23, 60).

Based on the pheWAS results, the largest effect is observed between impulsivity and suicidal ideation; with ADHD exhibiting the fourth largest effect. ADHD is a major public health concern that is often missed or misdiagnosed, especially in adults (61). Currently, one in nine children receive a diagnosis of ADHD in the U.S. (20), and the rates of ADHD diagnosis among adults are increasing (62). Furthermore, suicide has also been linked with impulsivity in the past (63), and higher impulsivity may have a role in the progression from ideation to attempt (64). The low number of participants reporting suicidal ideations and ADHD preclude further disease-free analysis at this time; however, as adult ADHD rates increase and Nevada currently ranks 12th in the nation for suicide rate, both observations require follow-up.

In addition to ADHD and suicidal ideation, other notable associations extend to chronic physical and mental health conditions, such as MDD and hypertension, both demonstrating significant positive effects with increasing levels of impulsivity. Considering that these major public health concerns can be captured by the HDSA, we propose integrating the HDSA into community health needs assessments. While self-assessments may be a useful tool for higher income individuals to make more informed healthcare decisions, that approach will likely neglect the most underserved who forego or delay care based on access and income; instead, solutions should involve integrating results with healthcare organizations such as Federally Qualified Health Centers (FQHC), to ensure those clinically underserved have access to the care they need. This holistic model of assessing the whole person is an approach being used at Northern Nevada HOPES, an FQHC in Reno/Sparks^1^, as well as the Reno-Sparks Community Health Alliance^2^. However, implementing even simplified assessments into clinical practice includes practical challenges for the patient intake assessments and clinician interpretation. Digital patient interfaces such as MyChart can help minimize time and effort for both parties. These resources, however, may not reach all patients, thus partnering with local FQHCs and community health organizations would improve access for at-need patients. Furthermore, statewide and national campaigns to focus on ACE and impulsivity assessment and prevention (similar to breast cancer screening campaigns) would help increase awareness for these more environmentally-driven disease risks.

Our study demonstrates two important relationships between impulsivity, ACEs, and the globally significant diseases MDD and hypertension (65, 66). First, the average age at diagnosis for both essential hypertension and MDD average was significantly different based on self-reported ACE score and/or impulsivity scale. A decade or more difference in disease onset is impactful on longer-term associated physical effects, quality of life (67), morbidity, mortality (68) and overall healthcare and economic costs. Second, both high ACEs and high impulsivity confer significant risk of MDD and essential hypertension for the general population. Despite adjusting for the combination of covariates: sex, ACEs, and impulsivity, a diagnosis of MDD is over twice as likely to occur for those with high ACEs and high impulsivity compared to those with no ACEs and low impulsivity. For essential hypertension, the diagnosis is approximately 1.25 times more likely to occur in those high groups. Essential hypertension accounts for 95% of all hypertension cases and is defined as high blood pressure in which secondary causes such as renovascular disease, renal failure, pheochromocytoma, aldosteronism, or other causes of secondary hypertension or mendelian forms (monogenic) are not present (69). It is also known as the ‘silent killer’ and often asymptomatic, but untreated can lead to stroke, kidney disease, vision loss, pulmonary hypertension, erectile dysfunction and more (70). Furthermore, we found that over an expected lifetime for both males and females, there is an approximately 40% difference in the predicted probability of being diagnosed with MDD between those with high ACEs and high impulsivity compared to those with no ACEs and low impulsivity. The difference in the predicted probability of essential hypertension diagnosis in the HNP_BIS_ is approximately 20%, though still statistically significant. The increased likelihood of hypertension decreases quality of life (71); it is also poorly managed in the general population (72) and we assume it is likely to be less managed in this high ACE and high impulsivity cohort. While the results are not surprising, they highlight the importance of considering a range of social health determinants, including socioeconomic status, health literacy, awareness, systemic factors that impact access to and ease of obtaining healthcare, drug use, alcohol use, dietary and tobacco habits (73, 74) when assessing patient risk factors.

## Conclusion

In summary, this study demonstrates the strong predictive capabilities of these two standard self-assessments regarding behavioral and mental health in the HNP_BIS_ population, consistent with prior studies (7, 38, 75). This suggests that the impact of life-history events such as ACEs and complex behavioral traits like impulsivity should be considered when assessing long-term risk of chronic diseases. Furthermore, we illustrate the potential risks of having severe childhood trauma and elevated impulsivity on the probability of being diagnosed with chronic disorders such as MDD and essential hypertension. When considered collectively, these findings highlight the potential for improving health outcomes through self-reported screenings. Given the utility of both the Barratt Impulsivity Scale (BIS-15) and ACE questionnaire as non-invasive, inexpensive diagnostic tools for adult health assessment, we advocate for their integration into routine patient care and broader population health studies.

### Strengths and contribution to existing knowledge

One of the unique components of the HNP_BIS_ is its range of longitudinal EHR data, which allows for time-to-event analysis. This enables us to measure lifetime hazards of participants’ health outcomes in combination with two major social health determinants. To the best of our knowledge, this type of survival analysis has not been performed. Additionally, the study presents the serious impact these two social determinants can have on both mental and physical health outcomes at various times during participants’ lifetimes. Furthermore, a full phenome-wide analysis yields an impressive overview of the impact of both impulsivity and childhood trauma. In the future, we propose to apply these methods to larger, diverse populations, and include other social health determinants as accessible proxies to disease. As a whole, this study adds to the growing body of literature describing the negative health outcomes associated with ACEs as well as increased impulsivity, and by applying time-to-event analysis, offers a long-term view of how early life stressors and behavioral traits influence specific disease progress.

### Limitations

Our study focused on self-reported impulsivity traits and childhood trauma occurrences, which are susceptible to possible self-reporting bias (76). To mitigate this bias, we validated ACE scores with previously published clinical associations that are related to ACEs (7). We also cross-referenced impulsivity scores with clinical associations in the pheWAS (Figure 1), observing many expected positive associations such as ADHD and suicidal ideation. The statistical models used in both the pheWAS and the disease-free analyses may be missing comorbidities that affect the outcome of disease and the predicted probability of disease. The male cohort in the HNP_BIS_ is smaller than the female cohort, which resulted in fewer observed events, particularly at older ages. This may affect the precision of the probability estimates for that cohort. Consequently, these findings should be interpreted with caution, especially when extrapolating to broader populations. Additionally, the HNP may not be a direct representation of the diversity of the general population. The HNP generally attracts educated, higher-income, female, Caucasian participants; a concerted effort is being made to increase HNP diversity by partnering with local Federally Qualified Health Centers.

## Data Availability

The datasets presented in this study can be found in online repositories. The names of the repository/repositories and accession number(s) can be found below: https://doi.org/10.5061/dryad.66t1g1k8x.

## Footnotes

^1^https://www.nnhopes.org/about/

^2^https://www.chanevada.org/about
