# Supplementary material for: A study of impulsivity and adverse childhood experiences in a population health setting
Source: Front Public Health. 2024 Dec 4;12:1447008. doi: 10.3389/fpubh.2024.1447008 (PMC11652370; doi:10.3389/fpubh.2024.1447008)
Supplement: Supplementary file 1 [file Data_Sheet_1.docx]

***Supplemental Methods***

*Data Disclosure Statement*

The data analyzed in this study are subject to the following licenses/restrictions: These data are available to qualified researchers upon reasonable request and with permission of the Center for Genomic Medicine. EHR data for the Healthy Nevada Project cohort are subject to HIPAA and other privacy and compliance restrictions. Phenotype data for each de-identified participant will be made available on https://datadryad.org/stash/share/NfPb4knSJY48LGb8qU8nfxIdupkUqZRlLEc1VrKWGZY, with a small subset of data removed to comply with HIPAA requirements.

The HNP encourages and collaborates with scientific researchers on an individual basis. Examples of restrictions that will be considered in requests to data access include but are not limited to: 1. Whether the request comes from an academic institution in good standing and will collaborate with our team to protect the privacy of the participants and the security of the data requested 2. Type and amount of data requested. 3. Feasibility of the research suggested. 4. Amount of resource allocation to support the collaboration. Any correspondence and data availability requests should be addressed to Joe Grzymski at (jgrzymski@med.unr.edu) or Craig Kugler (ckugler@med.unr.edu).

*The Renown EHR Database*

Renown EHR are based on the EPIC system (EPIC System Corporation, Verona, Wisconsin, USA). From these health records, we extract certain information regarding the overall health of Healthy Nevada Project (HNP) participants, including lab results, diagnosis codes, ICD codes (ICD9/ICD10) and specific demographic information. Currently, the EHR includes more than 1.7 million hospital patient visits between 2005 and 2024.

*Phenome-wide association studies*

The pheWAS, using the R package **pheWAS** as described in Carroll and Denny^1,2^, was based on the model

*PI ~ β_0_ + β_1_age + β_2_sex + β_3_BIS15 + β_4_ACEs*  (1)

Specifically, this model performs a logistic regression of disease/phenotype status (*PI=0,1*) versus the BIS-15 score for each of 1,252 phenotypes with more than 50 cases, controlling for ACEs, age and sex. The raw *p*-values for each of the 1,252 associations are shown in eTable 3 in Supplement 1; a Bonferroni correction for the number of comparisons provides *α* = 4 x 10^-5^ (0.05/1,252) as a significance threshold. The threshold is depicted by a horizontal red line in Figure 2.

***Supplemental References***

1. Carroll RJ, Bastarache L, Denny JC. R PheWAS: data analysis and plotting tools for phenome-wide association studies in the R environment. *Bioinformatics*. 2014;30(16):2375-2376. doi:10.1093/bioinformatics/btu197

2. Denny JC, Bastarache L, Ritchie MD, et al. Systematic comparison of phenome-wide association study of electronic medical record data and genome-wide association study data. *Nat Biotechnol*. 2013;31(12):1102-1111. doi:10.1038/nbt.2749

***Supplementary Table 1.* Questions for the Barratt Impulsivness Scale – short form (BIS-15)**

| **Questions** | **Subscale** |
| --- | --- |
| 1. I plan tasks carefully.* | non-planning |
| 2. I do things without thinking. | motor |
| 3. I don't pay attention. | attentional |
| 4. I concentrate easily.* | attentional |
| 5. I save regularly.* | non-planning |
| 6. I squirm at plays or lectures. | attentional |
| 7. I am a careful thinker.* | non-planning |
| 8. I plan for job security.* | non-planning |
| 9. I say things without thinking. | motor |
| 10. I act on impulse. | motor |
| 11. I get easily bored when solving thought problems. | attentional |
| 12. I act in the spur of the moment. | motor |
| 13. I buy things on impulse. | motor |
| 14. I am restless at lectures or talks. | attentional |
| 15. I plan for the future.* | non-planning |

* Asterisks indicate that these items are inversely scored. Responses to questions are: 1 = rarely/never, 2 = occasionally, 3 = often, 4 = almost always / always

***Supplementary Table 2.* Fisher exact test of high/low impulsivity and high/low ACEs**

|  | **High Impulsivity** | **Low Impulsivity** |
| --- | --- | --- |
| **High Aces** | 1441 | 2895 |
| **Low Aces** | 725 | 5181 |

* Odds ratio is 3.56; *p* < 2.2 x 10^-16^

** *Supplementary Table 3.* PheWAS Analysis stratified by ACEs, sex and age**

***Supplementary Table 4.* Disease-free survival for high impulsivity and low impulsivity for MDD**

|  | records | n.max | n.start | events | rmean | se(rmean) | median | 0.95LCL | 0.95UCL |
| --- | --- | --- | --- | --- | --- | --- | --- | --- | --- |
| LB | 6491 | 6491 | 6491 | 999 | 80.52 | 0.27 | NA | NA | NA |
| HB | 1451 | 1451 | 1451 | 454 | 68.37 | 0.75 | 70.22 | 67.03 | 73.06 |

* LB = Low Behavior; HB = High Behavior

***Supplementary Table 5.* Disease-free survival for high ACEs and no ACES for MDD**

|  | records | n.max | n.start | events | rmean | se(rmean) | median | 0.95LCL | 0.95UCL |
| --- | --- | --- | --- | --- | --- | --- | --- | --- | --- |
| NA | 4630 | 4630 | 4630 | 525 | 83.18 | 0.28 | NA | NA | NA |
| HA | 3312 | 3312 | 3312 | 928 | 70.95 | 0.51 | 74.32 | 72.16 | 78.64 |

* NA = No ACEs; HA = High ACEs

***Supplementary Table 6.* Hazard Ratios for MDD and essential hypertension stratified by High BIS-15, High ACEs and Sex**

| **Disorder** | **Model (Group)** | **Hazard Ratio** | **95% Confidence Interval** | **p-value** |
| --- | --- | --- | --- | --- |
| ***MDD*** | *HBIS* | 3.01 | 2.69-3.37 | 9.07E-83 |
|  |  |  |  |  |
| ***MDD*** | *ACEs* | 3.561 | 3.20-3.99 | 1.29E-116 |
|  |  |  |  |  |
| ***MDD*** | *HBIS+Sex (HBIS)* | 2.88 | 2.57-3.23 | 1.68E-76 |
| ***MDD*** | *HBIS+Sex (Sex)* | 0.43 | 0.38-0.50 | 3.46E-32 |
|  |  |  |  |  |
| ***MDD*** | *ACEs + Sex (ACEs)* | 3.25 | 2.92-3.63 | 2.43E-99 |
| ***MDD*** | *ACEs + Sex (Sex)* | 0.51 | 0.44-0.58 | 1.22E-21 |
|  |  |  |  |  |
| ***MDD*** | *HBIS+Sex+ACEs (HBIS)* | 2.17 | 1.93-2.43 | 1.78E-39 |
| ***MDD*** | *HBIS+Sex+ACEs (ACEs)* | 2.81 | 2.51-3.14 | 5.08E-72 |
| ***MDD*** | *HBIS+Sex+ACEs (Sex)* | 0.51 | 0.44-0.59 | 2.76E-21 |
|  |  |  |  |  |
|  |  |  |  |  |
| ***HyperT*** | *HBIS (<=25)* | 0.82 | 0.34-1.97 | 0.7 |
| ***HyperT*** | *HBIS (26-60)* | 1.35 | 1.20-1.52 | 5.8E-7 |
| ***HyperT*** | *HBIS (>60)* | 1.29 | 1.09-4.53 | 3.28E-3 |
|  |  |  |  |  |
| ***HyperT*** | *ACEs (<=25)* | 1.85 | 0.77-4.45 | 0.2 |
| ***HyperT*** | *ACEs (26-60)* | 1.22 | 1.10-1.35 | 1.55E-4 |
| ***HyperT*** | *ACEs (>60)* | 1.27 | 1.14-1.43 | 3.66E-5 |
|  |  |  |  |  |
| ***HyperT*** | *HBIS+Sex (HBIS <=25)* | 0.82 | 0.34-1.98 | 0.66 |
| ***HyperT*** | *HBIS+Sex (HBIS 26-60)* | 1.37 | 1.21-1.54 | 2.54E-7 |
| ***HyperT*** | *HBIS+Sex (HBIS >60)* | 1.32 | 1.11-1.56 | 1.41E-3 |
| ***HyperT*** | *HBIS+Sex (Sex <=25)* | 0.94 | 0.37-2.37 | 0.90 |
| ***HyperT*** | *HBIS+Sex (Sex 26-60)* | 1.26 | 1.13-1.40 | 3.18E-5 |
| ***HyperT*** | *HBIS+Sex (Sex >60)* | 1.22 | 1.09-1.35 | 4.04E-4 |
|  |  |  |  |  |
| ***HyperT*** | *ACEs + Sex (ACEs <=25)* | 1.86 | 0.77-4.50 | 0.17 |
| ***HyperT*** | *ACEs + Sex (ACEs 26-60)* | 1.27 | 1.15-1.41 | 5.97E-6 |
| ***HyperT*** | *ACEs + Sex (ACEs >60)* | 1.32 | 1.18-1.49 | 2.54E-6 |
| ***HyperT*** | *ACEs + Sex (Sex <=25)* | 1.02 | 0.40-2.59 | 0.97 |
| ***HyperT*** | *ACEs + Sex (Sex 26-60)* | 1.30 | 1.17-1.46 | 2.47E-6 |
| ***HyperT*** | *ACEs + Sex (Sex >60)* | 1.25 | 1.12-1.40 | 6.15E-5 |
|  |  |  |  |  |
| ***HyperT*** | *HBIS+Sex+ACEs (HBIS <=25)* | 0.74 | 0.30-1.78 | 0.50 |
| ***HyperT*** | *HBIS+Sex+ACEs (HBIS 26-60)* | 1.29 | 1.14-1.46 | 3.98E-5 |
| ***HyperT*** | *HBIS+Sex+ACEs (HBIS >60)* | 1.23 | 1.04-1.46 | 1.8E-2 |
| ***HyperT*** | *HBIS+Sex+ACEs (ACEs <=25)* | 1.95 | 0.80-4.75 | 0.14 |
| ***HyperT*** | *HBIS+Sex+ACEs (ACEs 26-60)* | 1.21 | 1.08-1.34 | 6.69E-4 |
| ***HyperT*** | *HBIS+Sex+ACEs (ACEs >60)* | 1.29 | 1.15-1.45 | 2.56E-5 |
| ***HyperT*** | *HBIS+Sex+ACEs (Sex <=25)* | 1.04 | 0.41-2.64 | 0.93 |
| ***HyperT*** | *HBIS+Sex+ACEs (Sex 26-60)* | 1.30 | 1.17-1.45 | 2.86E-6 |
| ***HyperT*** | *HBIS+Sex+ACEs (Sex >60)* | 1.26 | 1.13-1.40 | 4.12E-5 |

* HyperT = Essential Hypertension; HBIS = High BIS-15; ACEs = High ACEs; Sex = Sex coded 0 for female, 1 for male

***Supplementary Table 7.* Disease-free survival for high and low impulsivity stratified by sex for MDD**

|  | records | n.max | n.start | events | rmean | se(rmean) | median | 0.95LCL | 0.95UCL |
| --- | --- | --- | --- | --- | --- | --- | --- | --- | --- |
| LB, F | 4573 | 4573 | 4573 | 826 | 78.55 | 0.35 | NA | NA | NA |
| LB, M | 1918 | 1918 | 1918 | 173 | 84.73 | 0.39 | NA | NA | NA |
| HB, F | 1089 | 1089 | 1089 | 386 | 66.19 | 0.86 | 65.90 | 63.49 | 69.83 |
| HB, M | 362 | 362 | 362 | 68 | 75.68 | 1.45 | NA | 73.42 | NA |

* LB = Low Behavior; HB = High Behavior; M = Male; F = Female

***Supplementary Table 8*. Disease-free survival for high and low impulsivity stratified by sex and ACEs for MDD**

|  | records | n.max | n.start | events | rmean | se(rmean) | median | 0.95LCL | 0.95UCL |
| --- | --- | --- | --- | --- | --- | --- | --- | --- | --- |
| LB, F, NA | 2708 | 2708 | 2708 | 326 | 82.48 | 0.39 | NA | NA | NA |
| LB, F, HA | 1865 | 1865 | 1865 | 500 | 72.15 | 0.62 | 74.46 | 72.42 | 80.33 |
| LB, M, NA | 1454 | 1454 | 1454 | 99 | 86.29 | 0.36 | NA | NA | NA |
| LB, M HA | 464 | 464 | 464 | 74 | 77.83 | 1.02 | 87.46 | 84.30 | NA |
| HB, F, NA | 307 | 307 | 307 | 75 | 74.14 | 1.48 | 77.85 | 71.94 | NA |
| HB, F HA | 782 | 782 | 782 | 311 | 62.83 | 1.03 | 61.76 | 59.71 | 64.18 |
| HB, M, NA | 161 | 161 | 161 | 25 | 78.85 | 1.91 | NA | NA | NA |
| HB, M, HA | 201 | 201 | 201 | 43 | 72.75 | 2.17 | 75.86 | 72.12 | NA |

* LB = Low Behavior; HB = High Behavior; M = Male; F = Female; NA = No ACEs; HA = High ACEs

***Supplementary Table 9.* Disease-free survival for high and low impulsivity stratified by sex for essential hypertension**

|  | records | n.max | n.start | events | rmean | se(rmean) | median | 0.95LCL | 0.95UCL |
| --- | --- | --- | --- | --- | --- | --- | --- | --- | --- |
| LB, F | 4573 | 4573 | 4573 | 1528 | 69.00 | 0.32 | 69.39 | 68.84 | 70.16 |
| LB, M | 1918 | 1918 | 1918 | 873 | 67.29 | 0.40 | 68.00 | 67.18 | 68.68 |
| HB, F | 1089 | 1089 | 1089 | 374 | 63.16 | 0.72 | 63.16 | 61.69 | 65.57 |
| HB, M | 362 | 362 | 362 | 148 | 60.43 | 1.11 | 61.47 | 58.94 | 64.97 |

* LB = Low Behavior; HB = High Behavior; M = Male; F = Female

***Supplementary Table 10.* Disease-free survival for high and no ACEs stratified by sex for essential hypertension**

|  | records | n.max | n.start | events | rmean | se(rmean) | median | 0.95LCL | 0.95UCL |
| --- | --- | --- | --- | --- | --- | --- | --- | --- | --- |
| NA, F | 3015 | 3015 | 3015 | 1001 | 70.36 | 0.37 | 70.72 | 70.03 | 71.72 |
| NA, M | 1615 | 1615 | 1615 | 740 | 67.70 | 0.42 | 68.24 | 67.32 | 69.24 |
| HA, F | 2647 | 2647 | 2647 | 901 | 64.98 | 0.45 | 65.18 | 64.56 | 66.05 |
| HB, M | 665 | 665 | 665 | 281 | 63.00 | 0.82 | 63.49 | 61.52 | 64.99 |

* NA = No ACEs; HA = High ACEs; M = Male; F = Female

***Supplementary Table 11*. Disease-free survival for high and low impulsivity stratified by sex and ACEs for essential hypertension**

|  | records | n.max | n.start | events | rmean | se(rmean) | median | 0.95LCL | 0.95UCL |
| --- | --- | --- | --- | --- | --- | --- | --- | --- | --- |
| LB, F, NA | 2708 | 2708 | 2708 | 897 | 70.73 | 0.39 | 71.00 | 70.22 | 72.04 |
| LB, F, HA | 1865 | 1865 | 1865 | 631 | 66.13 | 0.52 | 66.09 | 65.35 | 67.21 |
| LB, M, NA | 1454 | 1454 | 1454 | 673 | 68.23 | 0.44 | 68.68 | 67.68 | 69.77 |
| LB, M HA | 464 | 464 | 464 | 200 | 64.37 | 0.95 | 64.29 | 62.48 | 66.54 |
| HB, F, NA | 307 | 307 | 307 | 104 | 66.27 | 1.25 | 68.06 | 65.99 | 71.77 |
| HB, F HA | 782 | 782 | 782 | 270 | 61.53 | 0.86 | 63.92 | 59.78 | 63.20 |
| HB, M, NA | 161 | 161 | 161 | 67 | 61.94 | 1.69 | 63.13 | 59.23 | 66.78 |
| HB, M, HA | 201 | 201 | 201 | 81 | 59.16 | 1.47 | 59.27 | 57.13 | 64.69 |

* LB = Low Behavior; HB = High Behavior; M = Male; F = Female; NA = No ACEs; HA = High ACEs

***Supplementary Figure 1.*** **Predicted probability of diagnosis stratified by high and low ACEs and sex for MDD**


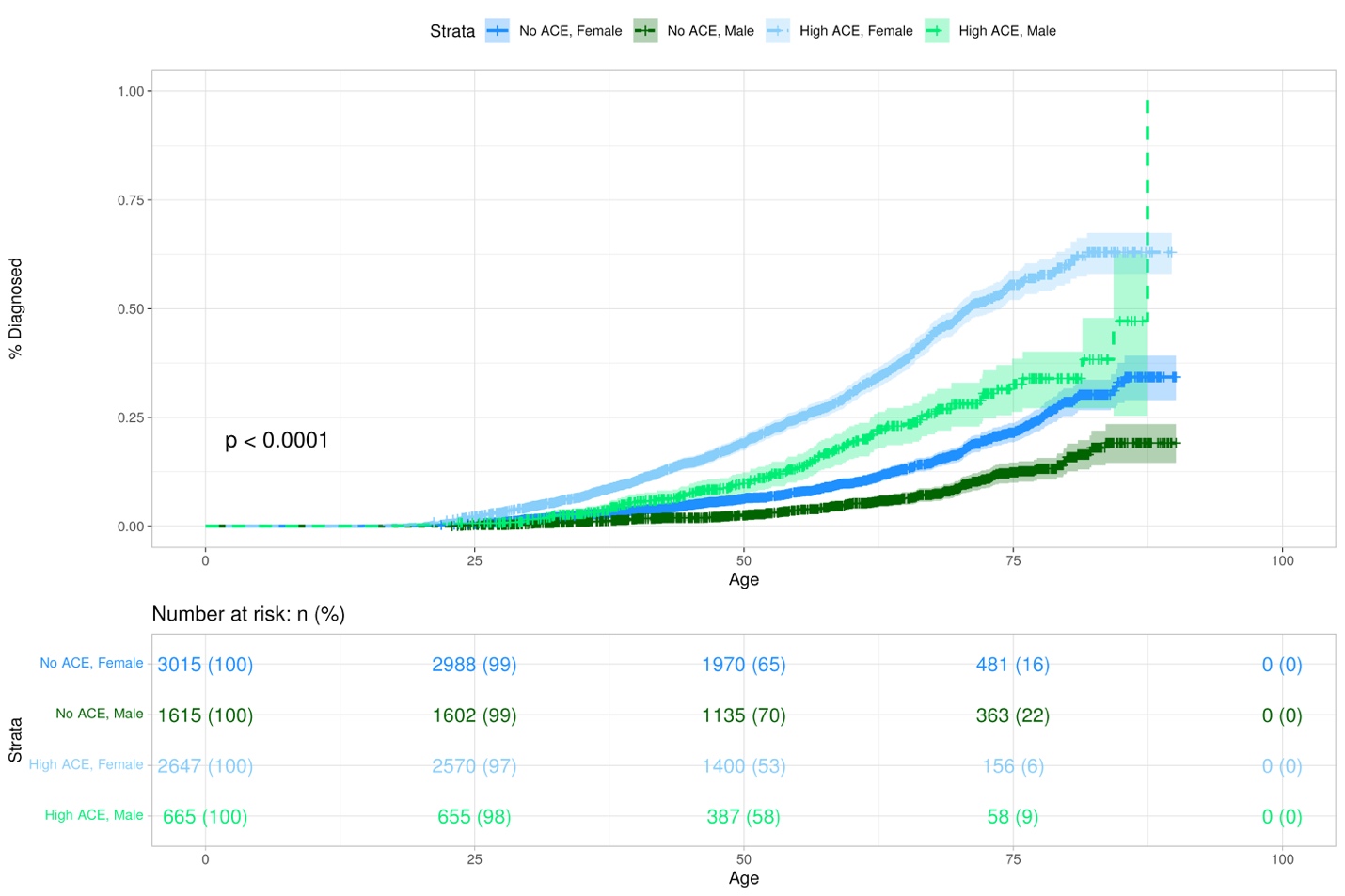


Supplementary Figure 1: This Kaplan-Meier plot demonstrates the predicted probability of MDD diagnosis over time for participants stratified by ACEs and sex. The y-axis represents the probability of being diagnosed with disorder. The x-axis represents participant age in years. Differences between the groups are visually depicted by the separation between the curves, indicating the relative rate at which events occur within each group. The shaded areas around each curve represent the 95% confidence intervals for the cumulative event estimates.

***Supplementary Figure 2.* Predicted probability of diagnosis stratified by high and low impulsivity and sex for essential hypertension**


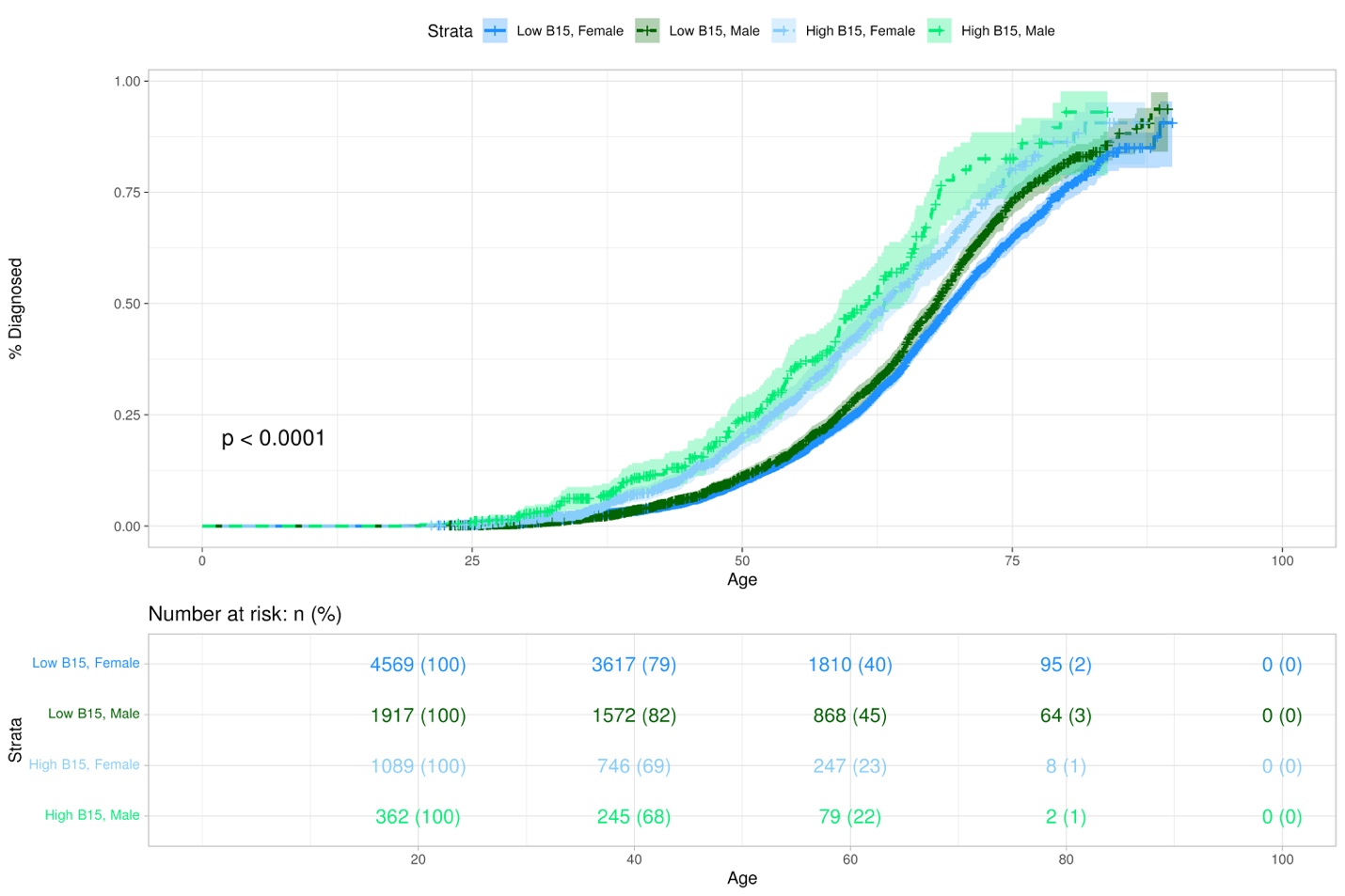


Supplementary Figure 2: This Kaplan-Meier plot demonstrates the predicted probability of essential hypertension diagnosis over time for participants stratified by impulsivity and sex. The y-axis represents the probability of being diagnosed with disorder. The x-axis represents participant age in years. Differences between the groups are visually depicted by the separation between the curves, indicating the relative rate at which events occur within each group. The shaded areas around each curve represent the 95% confidence intervals for the cumulative event estimates.

***Supplementary Figure 3.* Predicted probability of diagnosis stratified by high and low ACEs and sex for essential hypertension**

**
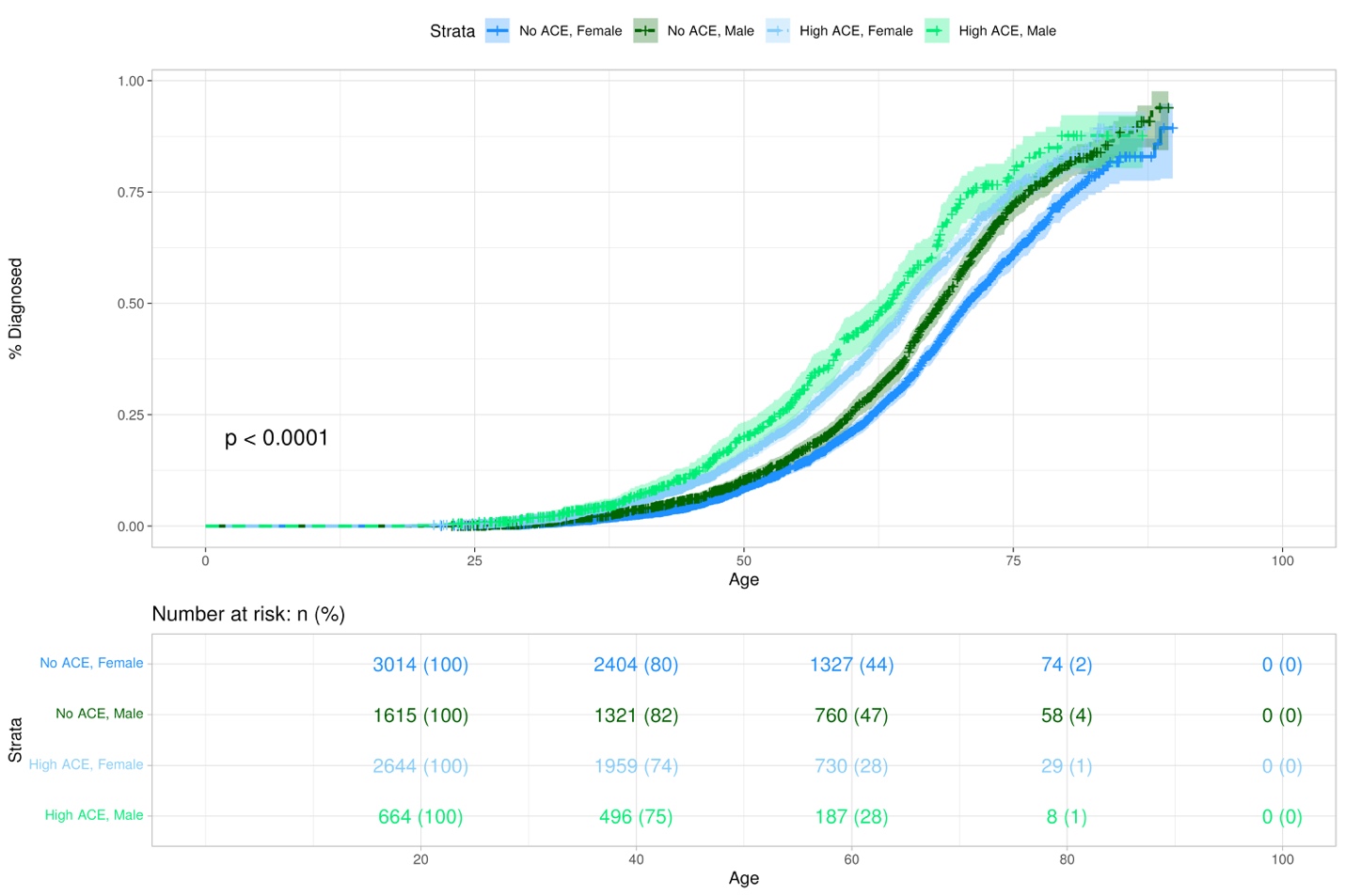
**

Supplementary Figure 3: This Kaplan-Meier plot demonstrates the predicted probability of essential hypertension diagnosis over time for participants stratified by ACEs and sex. The y-axis represents the probability of being diagnosed with disorder. The x-axis represents participant age in years. Differences between the groups are visually depicted by the separation between the curves, indicating the relative rate at which events occur within each group. The shaded areas around each curve represent the 95% confidence intervals for the cumulative event estimates.
